# Iatrogenic watering-can perineum and osteomyelitis of pubic ramus as a complication of post-urethral calculus removal UTI

**DOI:** 10.1259/bjrcr.20150045

**Published:** 2015-10-12

**Authors:** Kewal Arunkumar Mistry, Rohit Bhoil, Pokhraj Prakashchandra Suthar, Anurag Shukla

**Affiliations:** ^1^ Department of Radiology, Dr Rajendra Prasad Government Medical College, Kangra at Tanda, India; ^2^ Department of Radiology, Indira Gandhi Medical College, Shimla, India; ^3^ Department of Radiology, Medical College & SSG Hospital, Vadodara, India

## Abstract

We present a case of a 55-year-old male with a history of urethroscopic calculus removal who later developed urinary tract infection (UTI), complicated by periurethral abscess formation with osteomyelitis of the inferior pubic ramus and a urethrocutaneous fistula after surgical drainage of the abscess. UTI with periurethral abscess and urethrocutaneous fistula (watering-can perineum) is a rare complication of UTI. A periurethral abscess with pubic osteomyelitis has not been previously reported.

## Summary

Urinary tract infection (UTI) with periurethral abscess and urethrocutaneous fistula is a rare occurrence.^1^^–^^5^ Persistent urinary tract infections (UTI), indwelling catheters, undetected obstruction and trauma to the urethra are the common risk factors.^1^^–^^3^ Multimodality imaging is useful for accurate diagnosis. The abscess can be managed by drainage and the fistula can be treated by urethroplasty after an initial suprapubic cystostomy.^2,3,6–8^ An occurrence of periurethral abscess with pubic osteomyelitis has not been previously reported.

## Case report

**Figure 1.**
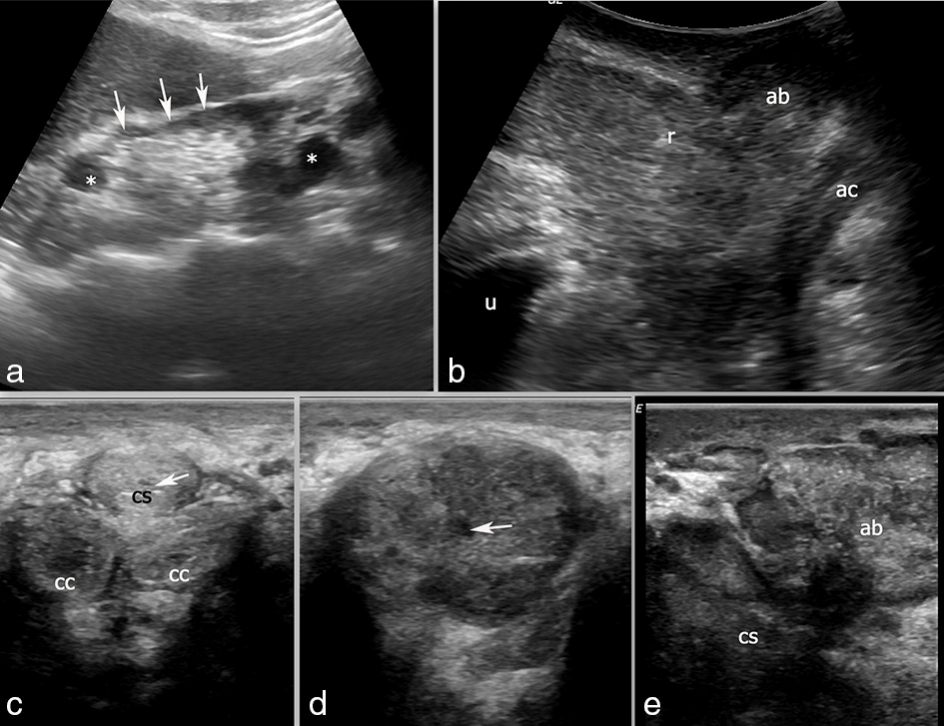
Pre-operative ultrasound images of the patient. (a) Longitudinal image of the left kidney showing cortical scarring (arrows) and caliectasis (asterisks). (b) Sagittal transperineal image shows an abscess (ab) in the perineum in relation to oedematous root of penis (r); the anal canal (ac) and urinary bladder (u) appear normal. (c) Axial image showing a normal proximal penile urethra (arrow) and normal parts of the corpus spongiosum (cs) and corpora cavernosa (cc). (d) Axial image showing a bulbar urethra (arrow) with an oedematous corpus spongiosum. (e) Sagittal transperineal image showing perineal abscess (ab) extending to the proximal corpus spongiosum (cs).

**Figure 2.**
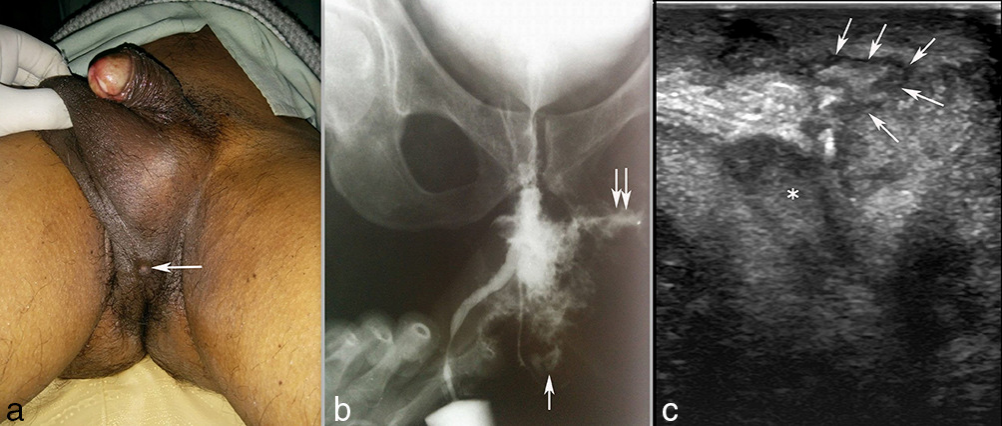
Post-operative clinical photograph (a) of the patient showing external opening of the fistula. Retrograde urethrogram (b) showing periurethral extravasation of the injected contrast with extension to the skin surface (arrow) and a lytic lesion in the left inferior pubic ramus (double arrow). Sagittal transperineal ultrasound image (c) showing the main fistulous tract (arrows) extending from the skin surface to the corpus spongiosum (asterisk).

**Figure 3.**
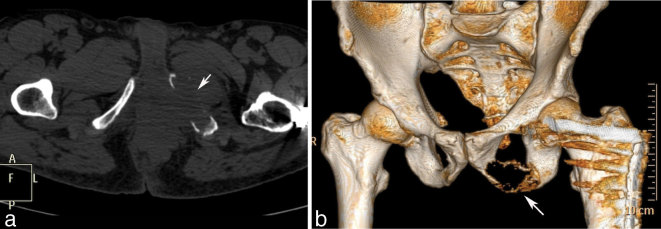
Post-operative axial (a) and volume-rendered (b) CT images showing a lytic lesion of the left inferior pubic ramus (arrows).

**Figure 4.**
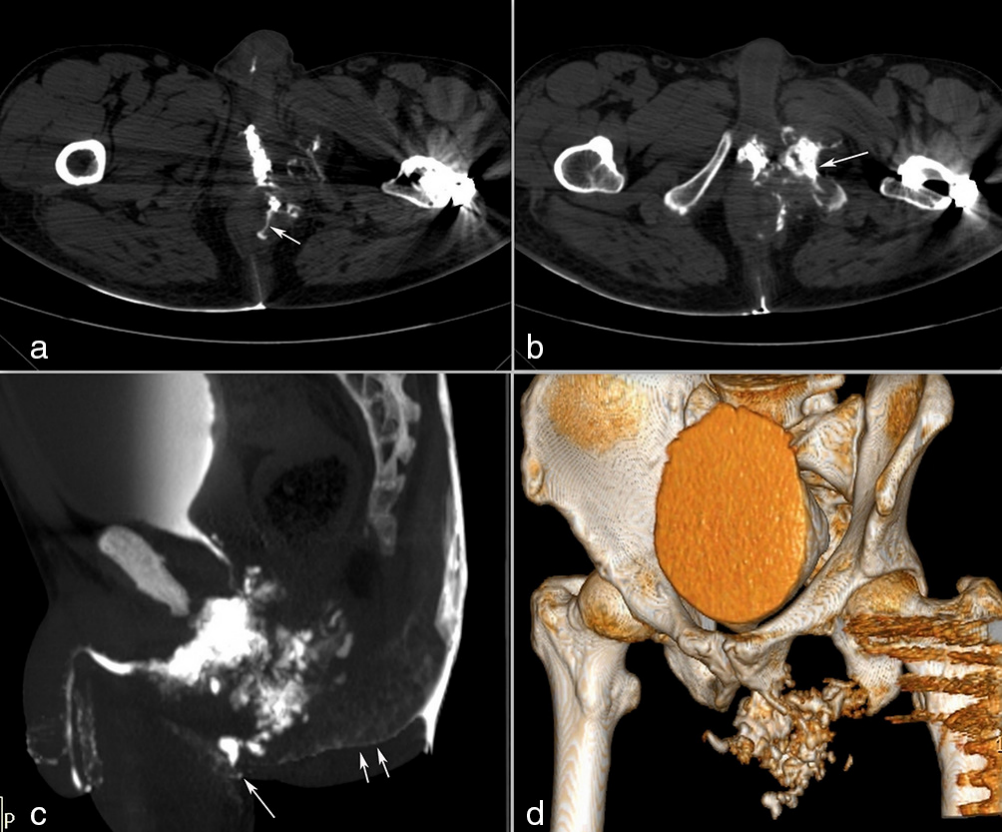
Post-operative axial retrograde CT urethrogram images (a and b) show extension of the injected contrast to intersphincteric planes of the anal canal (arrow in a) and a lytic lesion in the left inferior pubic ramus (arrow in b). The sagittal maximum intensity projection (c) and the volume-rendered (d) CT images show a complex periurethral collection communicating with the perineal skin (arrow in c) and spilling into the natal cleft (double arrow in c).

A 55-year-old non-diabetic, non-hypertensive male with a history of recurrent colicky left lumbar pain presented with acute urinary retention. Catheterization was attempted; however, it was unsuccessful. Ultrasound revealed an overdistended urinary bladder with a normal-sized prostate and scarring and focal caliectasis in the left kidney (Figure 1a). The right kidney was normal and no calculi were seen on either side on ultrasound. Serum electrolyte, renal and liver functions were normal. The haemogram revealed neutrophilia. The prostate-specific antigen was within normal limits. A rigid urethroscopy was performed owing to suspicion of a left urethral calculus and a 11-mm size calculus was removed from the posterior urethra. The patient was discharged and had no difficulty with micturition for 2 weeks thereafter. The patient subsequently developed burning micturition with hesitancy and induration in the perineal region. The urine was turbid and microscopy revealed the presence of *Escherichia coli.* A perineal ultrasound revealed an abscess in the perineum, which extended to the proximal parts of the corpus spongiosum (Figure 1b–e). This abscess was drained under saddle block. The patient subsequently developed a discharging sinus at the operative site (Figure 2a) leaking purulent fluid. A retrograde urography revealed periurethral extravasation of the injected contrast material with a lytic lesion in the left pubic ramus (Figure 2b). A repeat perineal ultrasound revealed a linear hypoechoic tract leading from the skin surface to the corpus spongiosum (Figure 2c). A retrograde CT urography was performed to look for the extent and ramifications of the abscess. On the non-contrast CT scan, a lytic lesion was seen involving the left inferior pubic ramus (Figure 3). The pubic symphysis and bodies of both pubic bones were normal. A proximal femoral nail was noted *in situ* on the left side, which was inserted 10 years before for fracture of the proximal shaft of the femur owing to accidental trauma. On injecting iodinated contrast into the urethra, there was extravasation of the contrast in the periurethral region in the soft tissues surrounding the bulbar and the posterior penile urethra. An extension of the contrast through the external anal sphincter into the intersphincteric plane (Figure 4a) with inflammatory stranding in the ischioanal fossae was seen. The contrast also extended into the lytic lesion present in the left inferior pubic ramus (Figure 4b). The contrast also extravasated through the cutaneous opening in the perineum and the natal cleft (Figure 4c,d). *E. coli* were isolated on pus culture. The patient was treated with intravenous antibiotics and suprapubic cystostomy was performed. The patient is presently being considered for elective urethroplasty.

## Discussion

In the past, before the advent of antibiotics, gonococcal urethritis was the most common cause of periurethral abscess.^1^^–^^3^ Presently, the most common microorganisms responsible for urethritis are Gram-negative rods, enterococci, anaerobes and, less often, staphylococci and streptococci, while *Staphylococcus epidermidis* and *Pseudomonas aeruginosa* have been implicated in immunocompromised patients.^1^^–^ ^4,9^ Risk factors for the development of periurethral abscess are persistent in UTI, indwelling catheters, undetected obstruction and trauma to the urethra.^2^^–^^5^ Uncommonly, penile cutaneous infections may be responsible.^1^ Urethral strictures, periurethral bulking agent injections, urethral diverticulae and urethral calculi have also been implicated as predisposing factors.^2,3^

Periurethral abscess results from rupture of the infected Littre’s glands, with extension of the infection into the submucosa. Rarely, such infection may attain sizes large enough to cause localized abscesses.^4^

Untreated periurethral abscess may progress to form single or multiple urethrocutaneous fistulae, the so-called *“watering-can perineum*”.^1^^–^^3^ These fistulae can be located in the perineum, scrotum, penis, penoscrotal junction and thighs. Initially, the patients are treated with suprapubic cystostomy followed by assessment of the stricture, and finally urethroplasty after a period of few months.^5^ The reported latent period between the original gonorrheal infection and the development of these sequelae can be a few months to 50 years.^6,9^ Emphysematous periurethral abscess has been reported in diabetic patients.^7,10^

Osteomyelitis of the pelvis is a rare condition and osteomyelitis of the pubic ramus without involvement of the body or symphysis of pubis has seldom been reported.^11^ A case of osteomyelitis of the pubic bone with vesicocutaneous and vesicovaginal fistula as a delayed complication of post-cervical cancer radiotherapy has been reported recently.^8^ To the best of our knowledge, there has been no report of periurethral abscess and urethrocutaneous fistula associated with pubic osteomyelitis.

## Learning points

1. In the past before advent of antibiotics, gonococcal urethritis was the most common cause of periurethral abscess.
2. Presently, the most common microorganisms responsible are Gram-negative rods, enterococci and anaerobes.
3. Risk factors are persistent UTI, indwelling catheters, undetected obstruction and trauma to the urethra.
4. Urethral strictures, periurethral bulking agent injections, urethral diverticulae and urethral calculi are other predisposing factors.
5. Periurethral abscess results from rupture of the infected Littre’s glands, with extension of the infection into the submucosa.
6. Untreated periurethral abscess may progress to form single or multiple urethrocutaneous fistulae, the so called *“watering-can perineum*”.
7. Watering-can perineum is a common sequelae of long-standing neglected inflammatory urethral stricture.
8. Osteomyelitis of the pelvis is a rare complication and osteomyelitis of the pubic ramus without involvement of the body or symphysis of pubis has seldom been reported.

## Consent

Informed consent to publish this case (including images and data) was obtained and is held on record.

## References

[1] Kraus S, Luedecke G, Ludwig M, Weidner W Periurethral abscess formation due to *Neisseria gonorrhoeae* . Urol Int 2004; 73: 358–60. 15604584 10.1159/000081600

[2] Komolafe AJ, Cornford PA, Fordham MVP, Timmins DJ Periurethral abscess complicating male gonococcal urethritis treated by surgical incision and drainage. Int J STD AIDS 2002; 13: 857–8. 12537744 10.1258/095646202321020189

[3] Sharfi AR, Elarabi YE The ‘watering-can’ perineum: presentation and management. Br J Urol 1997; 80: 933–6. 9439413 10.1046/j.1464-410x.1997.00487.x

[4] Blaschko SD, Weiss DA, Odisho AY, Greene KL, Cooperberg MR Proximal bulbar periurethral abscess. Int Braz J Urol 2013; 39: 137–8. 23489506 10.1590/S1677-5538.IBJU.2013.01.17

[5] Mungadi IA, Ntia IO Management of "watering-can" perineum. East Afr Med J 2007; 84: 283–6. 18254471

[6] Pandhi D, Reddy BSN Watering can perineum--a forgotten complication of gonorrhoea. J Eur Acad Dermatol Venereol 2002; 16: 486–7. 12428843 10.1046/j.1468-3083.2002.00652.x

[7] Ranjan P, Chipde SS, Prabhakaran S, Chipde S, Kapoor R Endoscopic management of emphysematous periurethral and corporal abscess. Niger Med J 2013 54: 209–10. 23900773 10.4103/0300-1652.114579 PMC3719251

[8] Salunke A, Nambi G, Manoharan A Osteomyelitis of the pubic bone with vesicocutaneous–vesicovaginal fistula: a delayed complication of post-cervical cancer radiotherapy. Niger Med J 2014 55: 83–5 24970977 10.4103/0300-1652.128179 PMC4071670

[9] Osoba AO, Alausa O Gonococcal urethral stricture and watering-can perineum. Br J Vener Dis 1976 52: 387–93. 1009418 10.1136/sti.52.6.387 PMC1045316

[10] Lee CY, Tsai HC, Lee SS, Chen YS Concomitant emphysematous prostatic and periurethral abscesses due to *Klebsiella pneumoniae*: a case report and review of the literature. Southeast Asian J Trop Med Public Health 2014 45: 1099–106. 25417511

[11] Al-Qahtani SM Osteomyelitis of the pubic ramus misdiagnosed as septic arthritis of the hip West Afr J Med 2004 23: 267–9. 15587845 10.4314/wajm.v23i3.28137

